# Highly Magnetized Encoded Hydrogel Microparticles with Enhanced Rinsing Capabilities for Efficient microRNA Detection

**DOI:** 10.3390/biomedicines9070848

**Published:** 2021-07-20

**Authors:** Wookyoung Jang, Jiwoo Kim, Seok Joon Mun, Sun Min Kim, Ki Wan Bong

**Affiliations:** 1 Department of Chemical and Biological Engineering, Korea University, Seoul 02841, Korea; wookyoung12321@gmail.com (W.J.); jjbaexx@korea.ac.kr (J.K.); msj95@naver.com (S.J.M.); 2 Seoul Metropolitan Government-Seoul National University Boramae Medical Center, Department of Obstetrics and Gynecology, Seoul 07061, Korea; sunmin827@hanmail.net; 3 Department of Obstetrics and Gynecology, Seoul National University College of Medicine, Seoul 03080, Korea

**Keywords:** magnetic hydrogel, microparticles, preeclampsia, microRNA detection

## Abstract

Encoded hydrogel microparticles mounting DNA probes are powerful tools for high-performance microRNA (miRNA) detection in terms of sensitivity, specificity, and multiplex detection capability. However, several particle rinsing steps in the assay procedure present challenges for rapid and efficient detection. To overcome this limitation, we encapsulated dense magnetic nanoparticles to reduce the rinsing steps and duration via magnetic separation. A large number of magnetic nanoparticles were encapsulated into hydrogel microparticles based on a discontinuous dewetting technique combined with degassed micromolding lithography. In addition, we attached DNA probes targeting three types of miRNAs related to preeclampsia to magnetically encoded hydrogel microparticles by post-synthesis conjugation and achieved sensitivity comparable to that of conventional nonmagnetic encoded hydrogel microparticles. To demonstrate the multiplex capability of magnetically encoded hydrogel microparticles while maintaining the advantages of the simplified rinsing process when addressing multiple samples, we conducted a triplex detection of preeclampsia-related miRNAs. In conclusion, the introduction of magnetically encoded hydrogel microparticles not only allowed efficient miRNA detection but also provided comparable sensitivity and multiplexed detectability to conventional nonmagnetic encoded hydrogel microparticles.

## 1. Introduction

MicroRNAs (miRNAs) are endogenous small fragments of noncoding RNAs that regulate gene expression by decreasing the accessibility of translation modules to mRNAs or through the degradation of mRNAs [1,2]. These miRNAs are abnormally dysregulated in various diseases such as cancer [3], Alzheimer’s disease [4], and viral infections [5]. miRNAs are known to be maintained in stable and reproducible forms in blood samples [6,7]. Accordingly, the detection of miRNAs by liquid biopsy has been widely performed for the successful and effective diagnosis of various diseases.

Considering the importance of miRNA detection for the diagnosis of various diseases, diverse techniques such as microarray detection [8], quantitative reverse transcription polymerase chain reaction (qRT-PCR) [9], northern blotting [10], and next-generation sequencing (NGS) [11,12] have been developed. However, these techniques have shortcomings in high-performance miRNA detection. For instance, the detection of short-length miRNAs is challenging with the microarray technique, which may be limited in the specific detection of multiple miRNA targets with similar sequences [13]. qRT-PCR lacks multiplex capability, owing to complicated primer design, and requires expensive costs [8,13]. In the case of northern blotting, detection sensitivity is low, and procedures are time-consuming [13]. Although NGS is capable of high-throughput and multiplexed detection with high sensitivity, there are still limitations, such as possibility of sequencing error and long process duration [11,12].

To overcome these limitations, poly(ethylene glycol) (PEG)-based geometrically encoded hydrogel microparticles synthesized by photopolymerization can serve as alternatives. These microparticles offer advantages such as multiplexing capability, high sensitivity and specificity, and short assay duration [14,15]. PEG components in hydrogel microparticles offer beneficial properties, such as an anti-fouling effect, high biocompatibility, tunability of mesh sizes, and convenient conjugation of functional molecules [16]. Geometrically encoded microparticles have distinct advantages, such as high encoding capacities and ease of code distinction as compared to other encoding methods, including the fluorescence spectrum and size of particles [17,18]. Graphical encoding can be easily achieved by photopolymerization of PEG-based hydrogel microparticles with various lithographic techniques, such as flow lithography [19], replica molding lithography [20], and contact lithography [21]. Considering these advantages, multiplexed miRNA detection based on graphically encoded hydrogel microparticles has been widely researched [22,23].

Despite the diverse merits of graphically encoded hydrogel microparticles for miRNA detection, significant limitations still exist. The need for multiple incubation steps during detection can be time-consuming but is imperative to completely exchange the incubation solution. As a proper tool for concise and rapid particle rinsing, magnetized microparticles allow magnetic separation during the rinsing process [24,25,26]. When a magnetic field is applied to a suspension composed of magnetic microparticles, particles can be immobilized in a certain area and the solvent can be easily exchanged without labor-intensive and repeated centrifugation steps. In addition, as magnetic separation does not require any direct contact between particles and external filters such as membrane filtration, the recovery process is simple and particle loss can be minimized. In particular, highly magnetized microparticles are advantageous for minimizing the loss of particles and reducing separation time by increasing the velocity of microparticles during magnetic separation [25].

Typical methods for producing magnetized graphically encoded hydrogel microparticles are based on the encapsulation of magnetic nanoparticles (MNPs) during particle synthesis [24,26,27,28] or post-synthesis modification of the particles [29]. In the encapsulation approach, MNP loading conditions during particle synthesis can be classified into flowing and static states of the precursor, such as microfluidics [24,26,27] and micromolding processes [28]. Micromolding-based MNP encapsulation offers remarkable advantages. It is capable of loading large amounts of MNPs during photopolymerization by exposing UV light to a static precursor, a process that is challenging with microfluidic lithography [30]. In addition, the vertical micropillar-based encoding method of micromolding is advantageous for high-resolution microparticles as compared to microfluidic processes, which suffer from the hardship of light focusing and excessive polymerization [28]. Although post-synthesis modification is favorable to produce highly magnetized microparticles, it has limitations, such as low particle resolution after modification and requirement of additional steps [29].

A discontinuous dewetting process on a degassed polydimethylsiloxane (PDMS) micromold was recently developed to produce highly magnetized encoded hydrogel microparticles [28]. This technique allows rapid precursor loading and prevents MNP separation when the precursor is squeezed between the mold and the substrate [28,31]. Particles synthesized using this method showed higher magnetization values than those reported in previous studies and better practical potential for biomarker detection in a singleplex protein immunoassay [28]. However, their applicability and detailed performance for miRNA detection have not been studied. In particular, their capability for multiplexed detection has not been proven, which is a critical factor for time-saving and cost-effective diagnosis [17].

In this study, we introduced an enhanced rinsing platform for efficient and rapid miRNA detection using highly magnetized encoded hydrogel microparticles, synthesized by discontinuous dewetting in a degassed PDMS mold. The saturation magnetization of the synthesized microparticles was 16.1 emu/g, which is higher than that reported in studies on MNP encapsulation with graphically encoded hydrogel microparticles. These highly magnetized microparticles were used to detect three types of miRNAs closely related to preeclampsia through simplified rinsing procedures using a magnetic separator. For high-performance miRNA detection, probes were attached to the particles via a post-synthesis functionalization method that utilizes click reactions between thiol groups of probes and unreacted acryl groups of microparticles, enabling high probe loading capacity [23]. We demonstrated that the detection limit was comparable to that in previous miRNA detection studies based on nonmagnetic hydrogel microparticles. Furthermore, triplex detection was performed to demonstrate the multiplexed detectability of magnetic-encoded hydrogel microparticles.

## 2. Materials and Methods

### 2.1. Materials

Sera-Mag magnetic carboxylate-modified particles (hydrophilic, 744 nm, 50 mg/mL) and Tris(2-carboxyethyl) phosphine (TCEP) were purchased from Thermo Fisher Scientific (Waltham, MA, USA). Poly(ethylene glycol) (MW 200 Da), poly(ethylene glycol) diacrylate (MW 700 Da), Darocur 1173 (2-hydroxy-2-methylpropiophenone), Tween 20, 1× phosphate-buffered saline (PBS), triethylamine (TEA), and streptavidin-phycoerythrin (SA-PE) were obtained from Sigma-Aldrich (St. Louis, MO, USA). Thiolated PEG 350 was supplied by Biochempeg (Watertown, MA, USA). The photoresist SU-8 25 master mold was procured from MicroChem (Newton, MA, USA). The PDMS ingredients SYLGARD 184 A/B were purchased from Corning (Corning, NY, USA). We prepared 1× Tris-ethylenediaminetetraacetic acid (EDTA) (1× TE) buffer from 100× Tris-EDTA (Sigma-Aldrich, USA) diluted in nuclease-free water (Thermo Fisher Scientific, USA). In addition, 10 mM adenosine 5-triphosphate (ATP), T4 DNA ligase, and NEBuffer 2 were obtained from New England Biolabs (Ipswich, MA, USA).

### 2.2. PDMS Mold Fabrication and Precursor Preparation

PDMS comprising 10:1 SYLGARD A and B (*v/v*) was thoroughly mixed and poured over the master mold. The hardened PDMS mold was cut away from the silicon wafer after baking it at 70 °C for 4 h. The height and surface area of the top of the mold were 3 mm and 1 cm^2^, respectively. The height of the microwell in the mold was 25 μm.

The hydrogel particle precursor liquid contained 75% (*v/v*) MNP suspension, 20% (*v/v*) PEG-DA, and 5% (*v/v*) Darocur 1173 photoinitiator. Before mixing with PEG-DA and Darocur 1173, the MNPs in the initial suspension were two-fold concentrated, and 75% (*v/v*) of the solvent was substituted with PEG 200 by removing supernatant after centrifugation. For the redispersion of MNPs, the microtube containing the MNP suspension was sonicated for 30 s.

### 2.3. Magnetic Particle Synthesis via Discontinuous Dewetting

The particles were prepared using a discontinuous dewetting method on a degassed mold developed in our previous study [28]. The PDMS mold was degassed (0.1 atm) for 10 min and placed on an Axiovert 200 microscope (Zeiss, Germany). The liquid precursor (12 μL) was dropped onto the degassed mold and covered with a cover glass. After the precursor was filled inside the microwell array, the cover glass was fixed at a certain position with a stand. The translational control knob of the microscope was attached to a 3D-printed gear and rotated at a steady pace as the connected gear motor spun, resulting in the PDMS mold sliding away from the cover glass. The rotation speed was regulated by LabVIEW software and the DAQ board (National Instruments, Austin, TX, USA). After dewetting, the precursor-loaded mold was placed in the degassed chamber for 10 min. Then, 365 nm UV light was irradiated at 200 mW/cm^2^ (Thorlabs, Newton, NJ, USA). To collect the synthesized particles, the mold was filled with 150 μL of PBS with 0.05% (*v/v*) Tween 20 (PBST) and frozen at −70 °C for 30 min. Frozen PBST with hydrogel magnetic particles attached underneath was placed in a microtube and rinsed five times with PBST.

### 2.4. Post-Synthesis Probe Conjugation

Thiolated probes were mixed with the same amount of 0.5 mM TCEP for reduction and maintained at 25 °C for an hour. Then, 10 μL of the reduced probe solution was incubated with 145 μL of PBST containing 6000 magnetic microparticles in a thermo shaker (Allsheng, Hangzhou, China) at 37 °C for 48 h at 1500 rpm. The particles were rinsed 10 times with 1× TE containing 0.05% (*v/v*) Tween 20 (1× TET).

To verify that the probes were attached to the unreacted acryl groups of the particles by the thiol-ene reaction, thiolated PEG 350 was used as a blocking agent. To block the treatment of unreacted acryl groups, particles dispersed in 95 μL PBST were mixed with 5 μL of thiolated PEG 350, and 4 μL of TEA was used as a catalyst. The mixture was then incubated at 37 °C for 6 h. These treated particles were redispersed in 145 μL PBST after rinsing five times with PBST. The particles were then reacted with thiolated probes using the post-synthesis probe conjugation method described above. Probes used in this step contained a 6-carboxyfluorescein (FAM) fluorescence tag at the 3′-end to measure the amount of probe loading. As a control, particles without blocking treatments were conjugated with FAM probes using the same procedure. Fluorescence signals were analyzed to compare the probe loading capacities between the blocked and unblocked particles.

### 2.5. Optimization of UV Exposure Time Corresponding to the Maximum Probe Loading Capacity

To find the optimal UV exposure time condition for maximizing the amount of probe loading in microparticles, we synthesized magnetic microparticles with various UV exposure time conditions from 200 to 1000 ms. After rinsing five times with PBST, DNA probes containing fluorescent 6-FAM dyes were attached by a post-synthesis reaction.

### 2.6. Magnetization Measurement and Calculation of Loading Capacity for MNPs

The magnetization of freeze-dried magnetic particles and MNPs was measured using a physical property measurement system (Quantum Design Inc., San Diego, CA, USA) containing a vibrating sample magnetometer (VSM). When a sample vibrates in a constant magnetic field, the magnetic flux changes. This change is detected by a coil near the sample and converted to digital signals. In this study, we applied magnetic fields ranging from −1.0 T to +1.0 T to acquire the magnetization values.

The loading capacity of the MNPs encapsulated in a unit hydrogel particle was calculated using the following equation:

(1) $$Loading\;Capacity=\frac{Mass\;of\;loaded\;MNPs}{Mass\;of\;a\;hydrogel\;particle}\times 100\;\left(\%\right)$$

### 2.7. Microplate-Based miRNA Detection Assay

The detection assay was performed in a 96-well plate for coordination with a simplified rinsing process based on a microplate-type handmade magnetic separator. miRNAs related to preeclampsia (miR-18a, miR-29a, and miR-210) were used in the current study as detection targets. First, the target miRNAs in a 1× TE buffer were diluted with the same buffer from 1 nM to 62.5 pM. Five microliters of the diluted target were then added to 10 μL of 1× TET (50 mM sodium chloride (NaCl)) and 30 μL of 1× TET, and incubated at 95 °C for 3 min. Then, 60 particles attached with probes dispersed in 5 μL of 1× TET buffer were added and the system was incubated at 55 °C for 1.5 h. The hybridization temperature in this study was frequently utilized in various encoded hydrogel microparticle based miRNA detection research studies [15,23,32,33,34,35]. The final salt concentration was 200 mM, which was the optimized value in our previous miRNA detection study for acquiring maximum signal intensities [23]. After incubation, the particles were rinsed thrice with 1× TET (50 mM NaCl).

A mixture comprising 6 μL of a universal adapter (10 μM), 3.75 μL of ATP (0.2 mM), 3.6 μL of T4 DNA ligase, 150 μL of NEBuffer 2 and 1350 μL of 1× TET was combined to connect the universal adapter with target-conjugated microparticles. The mixture (245 μL) was added to each well and incubated at 21.5 °C for 45 min. After incubation, the particles were rinsed thrice with 1× TET (50 mM NaCl). The final volume of the suspension after rinsing was adjusted to 50 μL.

To attach the fluorescence tag to the end of the universal adapter, 5 μL of a 10× diluted SA-PE solution was added to each well and incubated at 21.5 °C for 45 min. The particles were rinsed thrice with 1× TET (50 mM NaCl), and the final volume of the suspension was adjusted to 50 μL. All incubations were performed by covering the microwell with a sealing tape to minimize evaporation.

For multiplex detection of preeclampsia-related miRNAs, three types of probe-conjugated particles were added to each microwell (~60 particles per target). The final concentration of each target was 50 pM. Other assay conditions were the same as in singleplex detection.

### 2.8. Rinsing of Microparticles Based on Magnetic Separator

Particle rinsing during detection was carried out in a 96-well plate using a magnetic separator, as depicted in Figure S1. The neodymium magnets were attached to specific regions of the separator plate to immobilize magnetic microparticles at the corresponding points of the incubation plates following superimposition of the two plates. After incubation, the particles were localized and immobilized by the magnetic force of the neodymium magnets. Then, the residual solvent was diluted by repeatedly filling and removing the washing buffer.

### 2.9. Image Analysis of Microparticles

Grayscale fluorescence images of the microparticles were obtained using a CMOS camera (Prime, Tuscon, AZ, USA). Bright-field and RGB fluorescence images were obtained using an Eos 6D camera (Canon, Japan). The cameras were connected to the microscope to observe the particles. The fluorescence light was exposed with HXP 120V (Zeiss, Germany). Microscope filter sets of λ_ex_/λ_em_ = 450–490/515 nm and 546/590 nm were used for FAM and SA-PE analysis, respectively. The intensities of the fluorescence signals were measured using ImageJ software (1.53a, National Institutes of Health, Bethesda, MD, USA).

## 3. Results

### 3.1. Magnetized Encoded Hydrogel Microparticle Synthesis and Post-Synthesis Functionalization of DNA Probes

**Figure 1:**
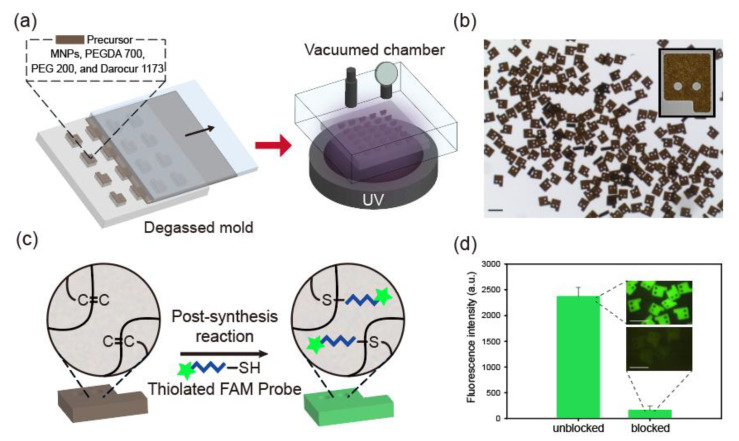
Overview of magnetic hydrogel microparticle synthesis and post-synthesis probe conjugation. (**a**) Scheme of discontinuous dewetting on a degassed PDMS mold. UV light is exposed in a vacuum chamber to offset the oxygen inhibition effect. (**b**) Bright-field images of synthesized magnetic microparticles. (**c**) Conjugation of thiolated FAM probes to magnetic microparticles via post-synthesis conjugation. (**d**) Fluorescence intensities of microparticles depending on blocking treatment for unreacted double bonds. Scale bars are 100 μm.

Magnetized encoded hydrogel microparticles were synthesized via a discontinuous dewetting process on a degassed PDMS mold, as illustrated in Figure 1a. To synthesize graphically encoded magnetic hydrogel microparticles, MNPs were incorporated into the precursor liquid comprising photoinitiator Darocur 1173 and the crosslinker PEGDA 700. Before loading the precursor onto the PDMS mold, the mold was degassed for rapid precursor loading by eliminating air bubbles through a gas-permeable PDMS structure [31]. The excess of precursor was removed through the horizontal movement of the cover slip, and the precursor suspension remained only in the micropatterned areas. Then, UV light was exposed in a vacuum chamber (0.1 atm) to suppress the oxygen inhibition effect from the surrounding atmosphere [36]. Within a second, high-resolution magnetic microparticles were uniformly produced, as shown in Figure 1b. Instead of PEG 600 used in a previous immunoassay study on magnetic encoded hydrogel microparticles, we selected PEG 200 as a porogen for the diffusion of the DNA probe and miRNA targets. PEG 200 as a porogen has been reported to effectively penetrate these small base-pair nucleic acids (<50 bp) in hydrogel microparticles [22,23].

After photopolymerization, the PEGDA monomer in hydrogel microparticles is abundant with unreacted double bonds owing to incomplete monomer conversion [37]. These unreacted double bonds enable attachment of DNA probes to the particles by chemical reaction for target-specific biomarker detection [23]. As depicted in Figure 1c, we conjugated thiolated DNA probes to unreacted double bonds in MNP-laden hydrogel microparticles by thiol-ene reaction to produce magnetized miRNA detection micromodules. The thiol-ene reaction is a well-known click chemistry that can occur under mild conditions at a high conversion rate [38].

To confirm that the DNA probes were loaded onto the microparticles following the reaction between their thiol groups and unreacted double bonds of the particles, we conjugated DNA probes containing fluorescent 6-FAM dyes to the microparticles where the unreacted double bonds were preliminarily blocked by thiolated PEG (SH-PEG) or left unblocked. The fluorescence intensity of the unblocked particles was 14.4 times higher than that of the blocked particles, implying that the unreacted double bonds of the particles were the key component in the probe conjugation process through enabling thiol-ene reactions (Figure 1d). Furthermore, this result indicates that the effect of nonspecific binding of nucleic acids to microparticles is negligible.

### 3.2. Characterization of Magnetization of Magnetic Encoded Hydrogel Microparticles

**Figure 2:**
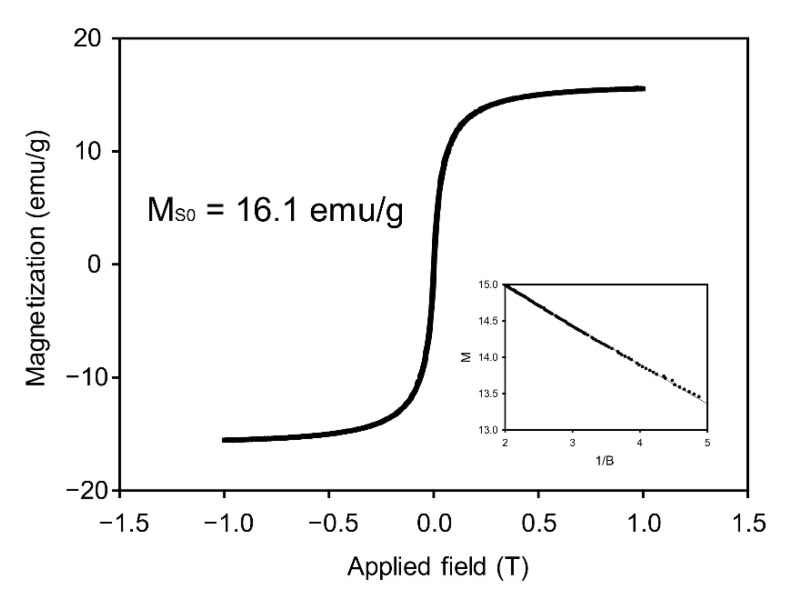
Magnetization value of magnetic hydrogel particles according to the intensities of an applied magnetic field. The inset is a linearized graph using the reciprocal of the magnetic field as the *x*-axis.

Practical suspension-based biomarker detection requires quick solvent exchange and stable particle immobilization for prompt diagnosis and stability of the overall process. Accordingly, the achievement of a high magnetization value of microparticles is important for process improvement and practical applications of hydrogel microparticle based miRNA detection. Thus, we measured the magnetization value of freeze-dried encoded magnetic hydrogel particles using a VSM (Figure 2). We calculated the saturation magnetization value of magnetic particles using the following equation [39]:

(2) $$M=M_{S0}\left(1-\frac{6}{\pi }\frac{k_{b}T}{M_{S}d^{3}B}\right)$$

M and M**_S0_** are the measured magnetization and saturation magnetization values of the particles, respectively, and B is the intensity of the magnetic field applied to the VSM. We plotted a linearized regression graph with the magnetization value of the particles (M) and reciprocal of the magnetic field intensity (1/B) and acquired saturation magnetization value as the y-intercept of the regression line. The saturation magnetization of magnetic hydrogel microparticles was 16.1 emu/g, which is, to the best of our knowledge, higher than that reported in previous studies on MNP encapsulation in geometrically encoded hydrogel microparticles [26,28]. This value is 1.9 times higher than that reported in our previous study, wherein magnetic-encoded hydrogel microparticles were synthesized in the same manner [28]. We speculate that PEG 200, which was used as a porogen in place of PEG 600, enabled efficient capturing of MNPs by decreasing the average pore size of the hydrogel microparticles [40].

The saturation magnetization value of lyophilized pure MNPs was 35.6 emu/g, which is 2.21 times the value observed for hydrogel microparticles (Figure S2). This ratio indicates that the weight composition of the polymer composites is 1.21 times that of MNPs in unit hydrogel microparticles because the magnetization of polymer composites is practically negligible. To quantitatively characterize the amount of MNPs loaded in hydrogel microparticles, we utilized a loading capacity equation that represents the mass ratio of MNPs encapsulated in hydrogel microparticles to total particles, including MNPs in dry conditions. Consequently, the loading capacity of MNPs in a single hydrogel microparticle was 45.2%.

### 3.3. Optimization of UV Exposure Time during Microparticle Synthesis for Optimum Probe Loading Density

**Figure 3:**
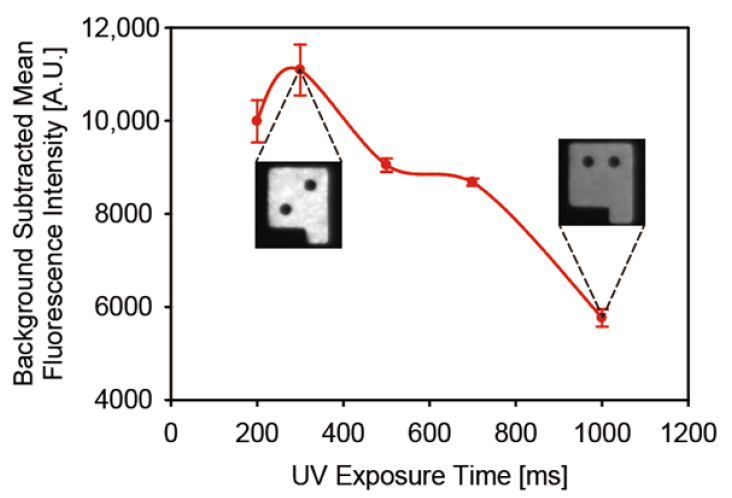
Probe loading densities in microparticles depending on UV exposure time.

To determine the optimal UV exposure time for maximum probe loading, we attached DNA probes containing 6-FAM dyes to encoded magnetic hydrogel microparticles and calculated the number of probes loaded on unit particles according to UV exposure time (Figure 3). The fluorescence intensity peak was observed at an exposure time of 300 ms, and a gradual decrease in signal intensity was noted after the peak point. We believe that the rapid increase in the unreacted double bonds before 300 ms was associated with the dominance of polymer chain propagation reaction over chain termination and oxygen inhibition reaction, owing to less conversion of monomers than of acryl bonds. In time ranges longer than 300 ms, the rate of propagation slowed down and the total amount of unreacted double bonds in microparticles started to diminish due to the steady consumption of the unreacted double bonds [37]. Only a UV exposure time shorter than a second is imperative for maximum probe loading onto encoded hydrogel microparticles despite the high UV absorption by MNPs in the precursor. This was possible owing to the low oxygen concentration in the degassed chamber, which could compensate for the UV absorption by MNPs during polymerization.

### 3.4. Singleplex miRNA Detection with Enhanced Rinsing Capabilities by Magnetic Separation

The efficient and prompt miRNA detection demands reduction in the detection duration and simplified procedures. To utilize the enhanced rinsing capability of highly magnetized encoded hydrogel microparticles for miRNA detection, we incubated and washed microparticles in 96-microwell plate with a microplate-based homemade magnetic separator, as described in a previous protein immunoassay procedure [28]. Detailed plate-based microparticle rinsing and miRNA detection procedures are illustrated in Figures S1 and S3. To evaluate the reduction in particle rinsing time through magnetic separation, we compared the rinsing duration with conventional centrifugation methods during the detection of a single miRNA target. The magnetic separation process reduced particle rinsing duration by 3.8 times as compared with centrifugation, from 38.9 to 10.3 min (Figure S4).

**Figure 4:**
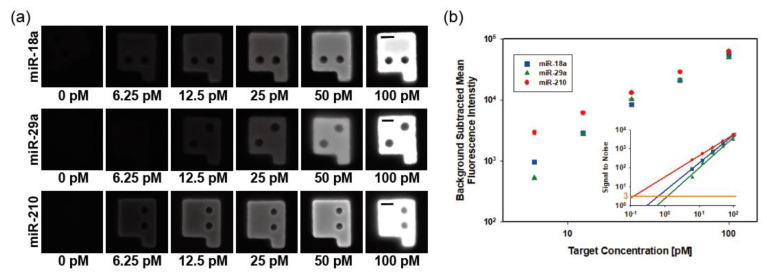
miRNA singleplex detection results using magnetic hydrogel microparticles. (**a**) Grayscale fluorescence images of particles observed during singleplex detection. Scale bars are 20 μm. (**b**) Background-subtracted fluorescence intensities of the particles. Inset is signal-to-noise ratio, signal values divided by corresponding standard deviations. LOD is the x value where each line intersects with y = 3.

To characterize the miRNA detection performance of magnetic-encoded hydrogel microparticles, we selected three types of miRNAs closely related to preeclampsia (miR-18a, miR-29a, and miR-210), and conducted singleplex detection for each target [41,42]. Target sequences are specified in Table S1. Preeclampsia is a complication of diverse pregnancy-related symptoms including hypertension, organ dysfunction, and proteinuria. Although its symptoms occur in the late stages of pregnancy, biomarkers of the disease are detectable in early stages from the first trimester [43]. Since preeclampsia is one of the major causes of maternal and perinatal death, early diagnosis and clinical treatment are necessary. As numerous biomarkers of preeclampsia are already discovered and multiplex detection of them enables precise diagnosis of diseases, preeclampsia fits the purpose of our research, which includes verifying the availability of detecting diverse biomarkers simultaneously. The fluorescence intensity of magnetic microparticles targeting each miRNA sequentially increased along with the target concentration from 6.25 to 100 pM (Figure 4a). To assess the assay performance of three preeclampsia-related miRNAs, we plotted the background-subtracted fluorescence intensities of microparticles versus target concentration (Figure 4b). In the range of selected standard concentrations, all fluorescence intensities for each target concentration represented linearized aspects. The inserted regression plot shown in Figure 4b indicates the signal-to-noise ratio along with the standard concentration of each spiked miRNA target. To evaluate the sensitivity for each target, we acquired the limit of detection (LOD) by calculating the target concentration that corresponds to three signals to noise. The LODs of miR-18a, miR-29a, and miR-210 were 33.3, 64.3, and 6.2 amol, respectively. These values, within an order, are comparable to those reported in previous studies on graphically encoded hydrogel microparticle-based miRNA detection [23].

### 3.5. Multiplexed Detection Performance of Magnetic Encoded Hydrogel Microparticles

**Figure 5:**
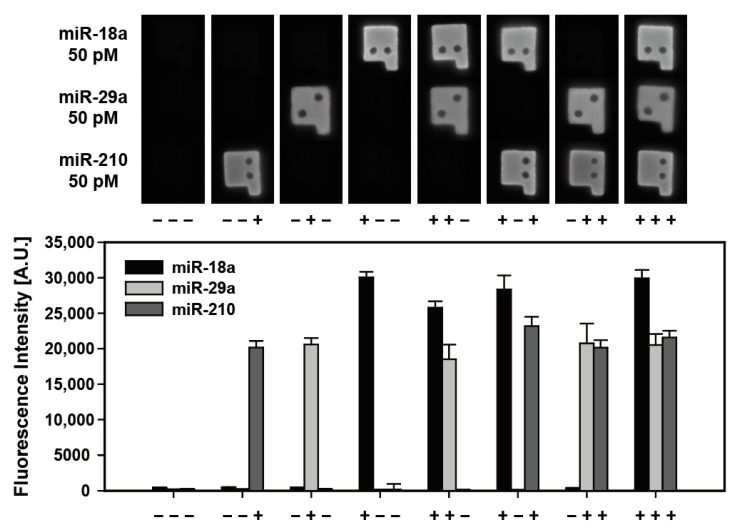
Multiplexed detection of three pre-eclampsia related miRNAs (miR-18a, miR-29a, and miR-210). The multiplexed detection was conducted for eight combinations of samples depending on the presence (+) or absence (−) of each target. The vertical bar graph indicates average fluorescence intensities of each target miRNA.

Simultaneous detection of multiple miRNA targets in a single sample is important for reliable and accurate disease diagnosis, high throughput, and cost reduction, as well as to minimize sample volume requirement [17]. Thus, to demonstrate multiplexed detectability of magnetically encoded hydrogel particles, we conducted a multiplexed detection of three preeclampsia-related miRNAs (miR-18a, miR-29a, and miR-210) each spiked at a 50 pM concentration into samples comprising eight combinations (Figure 5). The high resolution of graphically encoded magnetic microparticles made it possible to match the code of the particles with corresponding targets during observation. As shown in the particle fluorescence images for each combination and graph in Figure 5, there was no considerable cross-reactivity between each miRNA target. The fluorescence intensities of microparticles targeting the same species in different samples appeared at a similar level, which implies the capability of reproducible and specific multiplexed detection for samples of various compositions by magnetic-encoded hydrogel microparticles. The recovery rate, which is the ratio of the observed target concentration to the actual spike-in concentration, was 121.4%, 93.9% and 73.6% for miR-18a, miR-29a and miR-210, respectively. (Table S2) When the recovery rate is between 70–130%, it can be considered an acceptable process for industrial applications [44].

## 4. Discussion

In this study, we synthesized highly magnetized encoded hydrogel microparticles by a discontinuous dewetting technique using a degassed PDMS mold and applied them for multiplexed miRNA detection. This technique was rapid and involved simple particle rinsing via magnetic separation. Discontinuous dewetting on degassed PDMS molds enabled the production of highly magnetized sharp-edged microparticles, which were difficult to produce with typical flow lithography methods. The measured saturation magnetization of the microparticles was higher than that achieved in previous studies on MNP encapsulation within graphically encoded hydrogel microparticles. These highly magnetized microparticles decreased the particle rinsing time during detection by 3.8-fold as compared with the conventional centrifugation method.

In terms of assay performance, we optimized the UV exposure time during microparticle synthesis to achieve maximum probe loading density and performed miRNA detection of three preeclampsia-related targets. The fluorescence signals were linearly distributed according to the target concentrations, and the measured LOD was comparable (within an order) to that reported in previous hydrogel microparticle-based miRNA detection studies. To demonstrate the multiplexed detection potential of magnetic-encoded hydrogel microparticles, we conducted multiplexed miRNA detection with the same miRNA targets as in the singleplex detection assay. The magnetized encoded hydrogel showed high specificity and low cross-reactivity for each target, indicating no nonspecific adsorption between encapsulated MNPs and nucleic acids.

In summary, we introduced a rapid and efficient particle-rinsing platform for miRNA detection by encapsulating dense MNPs into hydrogel microparticles, demonstrating comparable sensitivity and multiplexed detectability to conventional nonmagnetic encoded hydrogel microparticles. We expect that the rinsing time will further decrease with a larger number of samples, especially during multiplexed detection.

## Supplementary Materials

The following are available online at https://www.mdpi.com/article/10.3390/biomedicines9070848/s1. Figure S1. Scheme of microparticle rinsing procedure via magnetic separation. Figure S2. M-H curve of lyophilized MNPs. Figure S3. Scheme for well plate based miRNA detection assay procedure in this study. Figure S4. Comparison of particle rinsing time during miRNA detection for single target between magnetic and centrifugal separation methods. Table S1. Sequences of DNA probes, miRNA targets and universal adapter. Table S2. Fluorescence signals of multiplexed detection of miR-18a, miR-29a, and miR-210.
