# Supplementary material for: Highly Magnetized Encoded Hydrogel Microparticles with Enhanced Rinsing Capabilities for Efficient microRNA Detection
Source: Biomedicines. 2021 Jul 20;9(7):848. doi: 10.3390/biomedicines9070848 (PMC8301431; doi:10.3390/biomedicines9070848)
Supplement: Supplementary file 1 [file biomedicines-09-00848-s001.zip › biomedicines-1291035-sm final.pdf]

Supplementary Information

Highly Magnetized Encoded Hydrogel Microparticles with

Enhanced Rinsing Capabilities for Efficient microRNA Detection

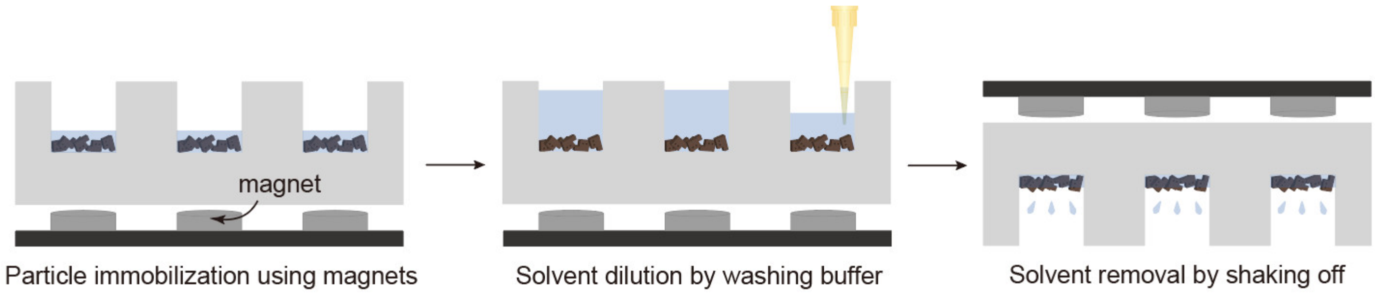

Figure S1. Scheme of microparticle rinsing procedure via magnetic separation. After incubation, particles are localized and immobilized by a homemade magnetic separator. Next, washing buffer is added to dilute the solution. Then, solvent is removed by shaking off the microplate. Above procedures are repeated for intended dilution rate.

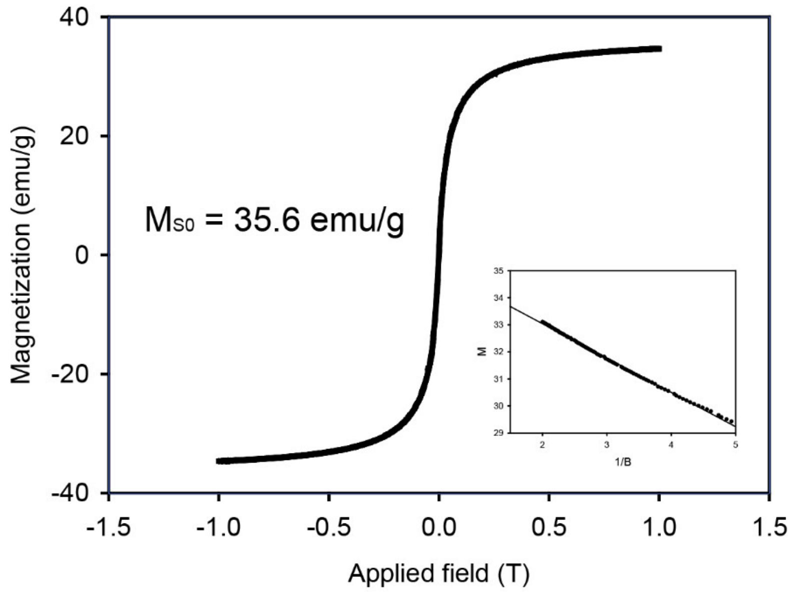

Figure S2. M-H curve of lyophilized MNPs. Saturation magnetization was calculated by the same manner as magnetic encoded hydrogel microparticles.

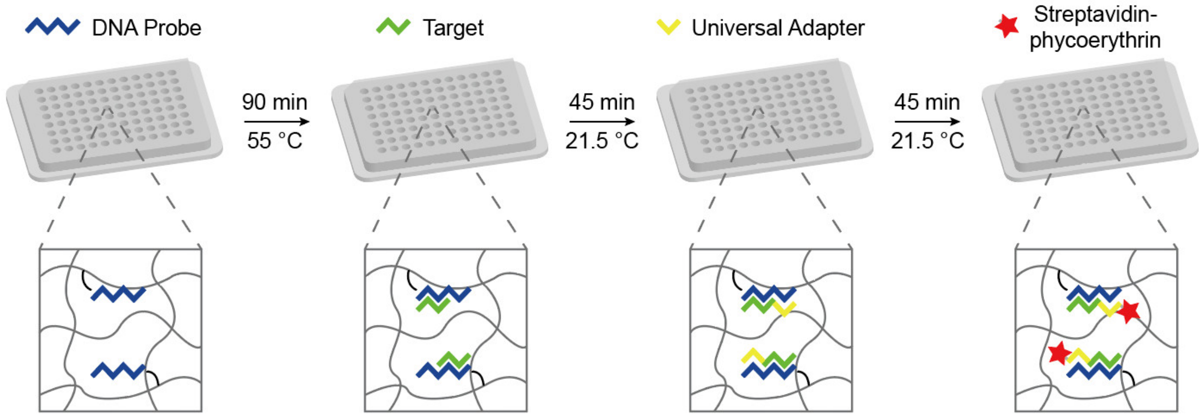

Figure S3. Scheme for well plate based miRNA detection assay procedure in this study. First, probe conjugated magnetic microparticles are hybridized by incubating with miRNA targets. Then, biotinylated universal adapters are attached by ligation via T4 DNA ligase. After universal adapter attachment, fluorescent streptavidin-phycoerythrin is conjugated to the universal adapter by streptavidin-biotin interaction for fluorescence labelling.

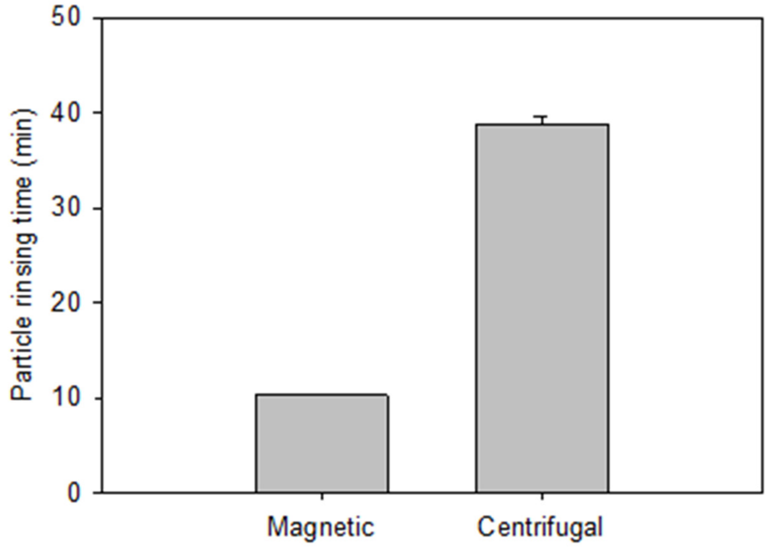

Figure S4. Comparison of particle rinsing time during miRNA detection for single target between magnetic and centrifugal separation approaches. Measurements were conducted in triplicate.

Table S1. Sequences of DNA probes, miRNA targets and universal adapter. Red-colored bases mean universal adapter sequences.

Name		Sequence
miR-18a-5p	Target	5′-UAA GGU GCA UCU AGU GCA GAU AG-3′
	Probe	/5ThioMC6-D/GAT ATA TTT TAC TAT CTG CAC TAG ATG CAC CTT A/3InvdT/
miR-29a-3p	Target	5′-UAG CAC CAU CUG AAA UCG GUU A-3′
	Probe	/5ThioMC6-D/GAT ATA TTT TAT AAC CGA TTT CAG ATG GTG CTA/3InvdT/
miR-210-3p	Target	5′-CUG UGC GUG UGA CAG CGG CUG A-3′
	Probe	/5ThioMC6-D/GAT ATA TTT TAT CAG CCG CTG TCA CAC GCA CAG/3InvdT/
Universal adapter		/5Phos/TAAAATATATAAAAAAAAAAAA/3Bio/

Table S2. Fluorescence signals of multiplexed detection of miR-18a, miR-29a, and miR-210.

Case		miR-18a		miR-29a		miR-210
1	−	462.2 ± 19.10	−	215.6 ± 24.40	−	256.6 ± 13.34
2	−	492.3 ± 26.24	−	222.2 ± 12.76	+	20155.1 ± 926.4
3	−	469.6 ± 21.44	+	20600.3 ± 904.62	−	229.8 ± 57.64
4	+	30049.9 ± 786.2	−	150.2 ± 6.07	−	172.2 ± 9.75
5	+	25775.2 ± 891.2	+	18504.6 ± 2068.2	−	139.7 ± 11.86
6	+	28355.8 ± 1962.0	−	131.4 ± 9.95	+	23179.3 ± 1322.1
7	−	398.4 ± 11.71	+	20757.0 ± 2788.3	+	20146.8 ± 1057.4
8	+	29913.5 ± 1188.1	+	20514.8 ± 1551.3	+	21572.4 ± 955.5
AVG		28391.0 ± 2157.5		20094.2 ± 2027.5		21263.4 ± 1618.2
Recov.(%)		121.4		93.9		73.6

Averages were calculated only with the signal values of target-present (+) ones. Recoveries are the ratio of the observed target concentration to the actual spike-in concentration.
